# Combination of orthokeratology lens with 0.01% atropine in slowing axial elongation in children with myopia: a randomized double-blinded clinical trial

**DOI:** 10.1186/s12886-022-02635-0

**Published:** 2022-11-15

**Authors:** Shiao Yu, Liping Du, Na Ji, Binbin Li, Xuena Pang, Xiuhong li, Nana Ma, Congcong Huang, Aicun Fu

**Affiliations:** 1 The First Affiliated Hospital of Zhengzhou University, 450000 Zhengzhou, China; 2 The Affiliated Eye Hospital of Suzhou Vocational Health College, 215000 Suzhou, China

**Keywords:** Orthokeratology, 0.01% atropine, Additive effects, Axial length, Myopia

## Abstract

**Background:**

To evaluate the additive effects of orthokeratology (OK) lenses and 0.01% atropine on slowing axial elongation in myopic children.

**Methods:**

A prospective, randomized, double-blinded, placebo-controlled trial was conducted over a 12-month period. Sixty children aged 8 to 12 years with spherical equivalent refraction from − 1.00 to -4.00 D who had been wearing OK lenses successfully for 2 months (as baseline) were randomly assigned in a 1:1 ratio to combination group (combination of OK lens and 0.01% atropine eye drops) and control group (combination of OK lens and placebo). The primary outcome was change in axial length, along with secondary outcomes including change in pupil diameter (PD) and accommodative amplitude (AMP) at 12 months (measured at 4-month intervals).

**Results:**

After 12 months, the overall axial elongation was 0.10 ± 0.14 mm and 0.20 ± 0.15 mm (p = 0.01) in the combination and control groups, respectively. The change in axial length in the two groups showed significant differences only in the first four months (median [Q1, Q3] (95% CI), -0.01 mm [-0.07, 0.05] (-0.06, 0.04) vs. 0.04 mm [0.00, 0.10] (0.02, 0.09); p = 0.04), but no difference thereafter. Multivariate linear regression analysis showed that the axial elongation was significantly slower in the combination group than in the control group (standard β = -0.10, p = 0.02). PD significantly increased by 0.45 mm [0.20, 0.68] at the 4th month visit (p < 0.001) and then remained stable in the combination group. The PD in the control group and AMP in the two groups remained stable from baseline to 12 months (all p > 0.05).

**Conclusion:**

The combination therapy was more effective than the OK lens alone in slowing axial elongation after 12 months of treatment, and mainly in the first 4 months.

**Trial registration:**

The First Affiliated Hospital of Zhengzhou University, ChiCTR2000033904. Registered 16/06/2020, http://www.chictr.org.cn/login.aspx?referurl=%2flistbycreater.aspx

## Background

The prevalence of myopia, including high myopia, has significantly increased worldwide, especially in the young age group of East Asia [1]. The increased severity of myopia significantly increases the risk of irreversible vision loss. Currently, orthokeratology (OK) and low- concentration atropine eye drops are commonly used to slow myopia progression [2–7]. OK lens could temporarily reduce myopia severity by reshaping the cornea, and OK control myopia progression most likely by increased peripheral myopic focus that reduces stimuli for axial elongation and subsequent development of myopia [8–10]. Atropine is a non-selective antagonist of muscarinic acetylcholine receptors that may mediate axial elongation by blocking of the muscarinic receptors in the retina and sclera [11, 12]. Many studies have shown that some concentrations of low concentration atropine can effectively control the progression of myopia [5–7, 13]. Conversely, it has been observed that the efficacy of the OK lens and low concentrations atropine was associated with many factors [14, 15], and that OK lens and low concentration atropine failed to control myopia progression in a small number of children.

To date, several clinical trials for over 1 year [16–19] and meta-analyses [20–22] have reported that low concentrations of atropine combined with an OK lens are more effective in controlling axial elongation than using an OK lens alone for children with myopia. The most recent meta-analysis included 8 studies [20]. However, of these 8 studies, two studies included only 1-month results and one study included 6-month results. Studies found that the combination therapy was more effective than the OK lens alone only in the first few months, but not different in the later stages of the combination [17, 18]. In addition, the study design of all these clinical trials were not randomized double-blinded, placebo-controlled studies, but rather randomized not-blinded, retrospective, or non-parallel studies [16–19]. In general, a blinded trial is regarded as being less subject to bias than an open trial because it minimizes the impact of knowledge of treatment allocation on post-randomized treatment decisions and on reporting of outcomes [23]. Therefore, in this study, a randomized, double-blinded, placebo-controlled one-year trial was conducted to evaluate the additive effects of OK lens and 0.01% atropine on slowing axial elongation in central mainland Chinese children with myopia. Axial elongation, accommodative amplitude (AMP) and pupil diameter (PD) were compared between children using OK lenses only and those using combination treatment, placebo is used in the OK lenses group only.

## Methods

### Study design

**Fig. 1:**
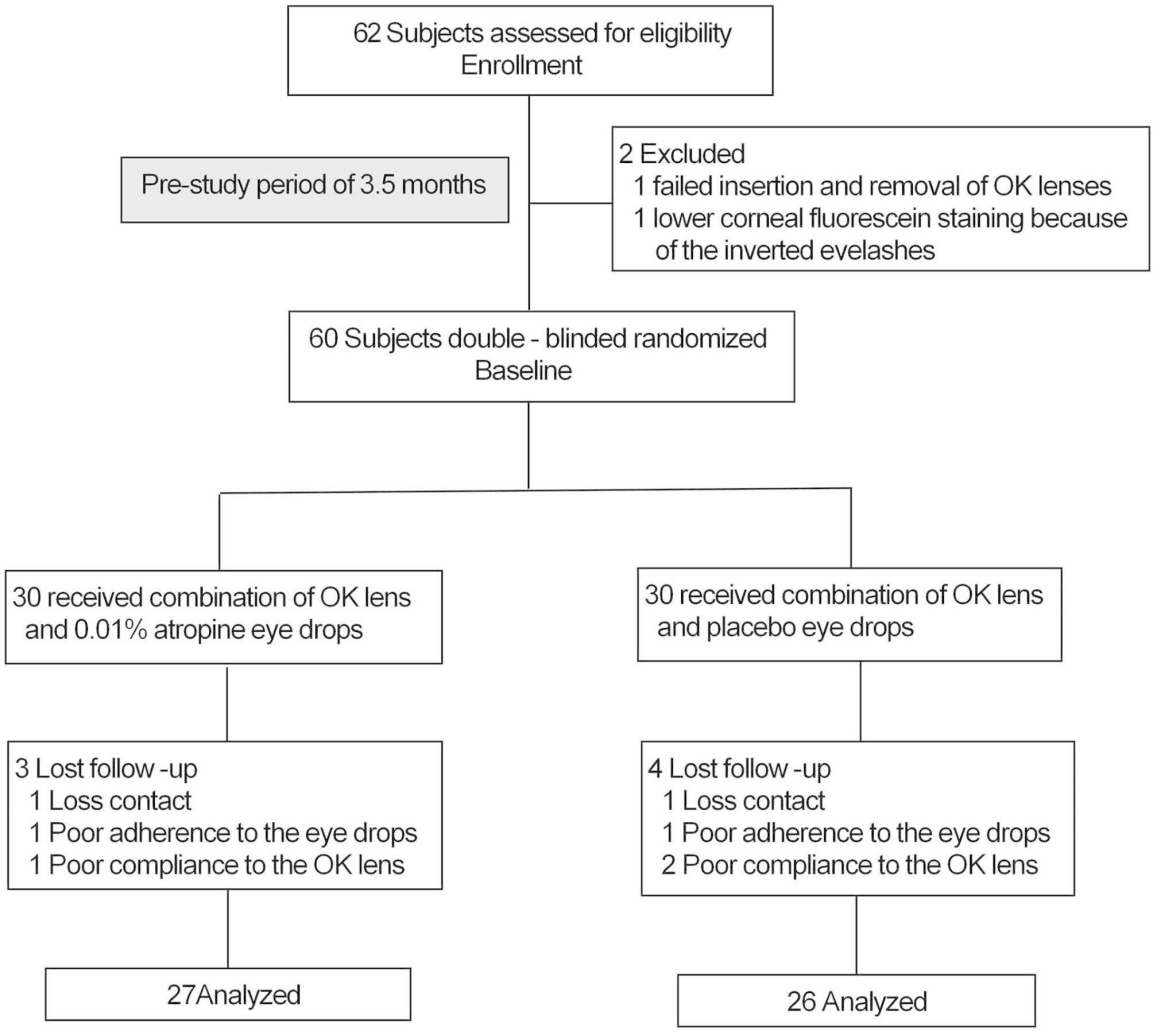
Subject recruitment and randomization flowchart

This study was conducted in two phases. All children who had been wearing the OK lenses successfully for 2 months were randomly assigned in a 1:1 ratio to the combination group (combination of OK lens and 0.01% atropine eye drops) and the control group (combination of OK lens and placebo) for 1 year in phase 1 (Fig. 1). At the beginning of the second year in phase 2, the combination group would be crossed over to the control group, and the control group would be crossed over to the combination group. The current study reported the 1-year phase 1 results. This trial was approved by the Human Ethics Committee of the First Affiliated Hospital of Zhengzhou University (Number: 2020-KY-223) and registered in the Chinese Clinical Trial Registry. This study conformed to the tenets of the Declaration of Helsinki. Written informed consent was obtained from the parents or guardians before the procedures, and the possible risks were fully explained before treatment initiation.

### Study Population

Sixty Chinese myopic children (Han nationality) who visited the First Affiliated Hospital of Zhengzhou University between June 2020 and September 2020 were recruited for this trial. The inclusion criteria were: 8–12 years of age, cycloplegic spherical equivalent refraction (SER) from − 1.00 to -4.00D, astigmatism less than 1.50D, anisometropia of less than 1.00D, monocular best corrected visual acuity of 20/20 or better, intraocular pressures not greater than 21 mmHg, no other eye diseases and surgery, no eye and systemic organic changes affecting vision acuity. The exclusion criteria were as follows: poor OK lens fit, congenital myopia (myopia present at birth) and pathological myopia, preterm birth, low-weight infants at birth, inability to comply with the study visit schedule, previous use of atropine or pirenzepine, peripheral defocus contact lenses and spectacles, and OK lenses to control myopia progression.

### Randomization and masking

A stratified block randomization method was used to control for age and SER at baseline (2 months after wearing lenses). Specifically, children were divided into four subgroups, by age (8.0–10.0 years and 10.1–12.0 years) and by SER (-1.00 to -2.50 D and − 2.51 to -4.0 D), then, the children in each subgroup were randomly allocated into combination and control groups.

The 0.01% atropine and placebo eye drops were packaged in identical bottles; thus, neither the examiner nor the participants were able to identify the contents. All the eye drops were maintained and distributed by the same doctor. The data analysts were blinded to reduce observational bias.

### Eye drops and OK lenses

The 0.01% atropine eye drops (pH = 5.4–5.6, 3 mL sealed bottle, 15–25 °C room temperature storage, discarded eye drops after opening the bottle for 1 month) were prepared by diluting atropine sulfate powder (Shaoxing Minsheng Medical Co., Ltd., Zhejiang, China) with normal saline under sterile conditions and subsequently, adding a preservative (0.3 mg/mL ethylparaben). The 0.01% atropine eye drops showed minimal degradation (approximately 1.8%) after opening the eye drop bottle for one month, and its properties were stable. A blank solvent without atropine was used as a placebo eye drops. All children were instructed to use 0.01% atropine or placebo eye drops by instilling one drop in both eyes once nightly 10 min before OK lens insertion.

The OK lenses used in this trial were four-zoned reverse-geometry lenses (Boston EM Material, Alpha Corp., Nagoya, Japan) with a Dk of 100 × 10^–11^ cm^2^/s (mL/O^2^/mL × mmHg). All children were fitted with lenses using trial lenses by the same ophthalmologist focusing on optometric eye care, according to the manufacturer’s instructions. The patients were advised to wear the lenses every night for at least 8 consecutive hours. The patients were also required to attend routine aftercare (1 day, 1 week, 2 months, and every 4 months after lens delivery) and unscheduled visits whenever necessary to ensure good ocular response and health. Lenses were routinely replaced about every 1.5 years, but if the residual SER was found to be more than 0.50 D at any visit after stabilization of treatment, lenses would be reordered. As the central corneal thickness stabilizes after wearing the OK lens for 1–2 months [24, 25], axial length (AL) measurement after wearing the OK lens for 2 months was used as the baseline value. Further, due to disruptions caused by COVID-19, with long inspection time of imported goods and slow delivery speed, it would take the participants approximately 1.5 months from fitting to wearing the OK lens. Therefore, the children in the two groups started to use 0.01% atropine or placebo once nightly at approximately 3.5 months (as baseline) after enrollment. All adverse events were recorded.

Each child was given four bottles of eyedrops at the randomization visit and the subsequent 4 monthly follow-up visits. These four bottles of atropine were collected at follow-up visits to track and monitor children’s compliance. Compliance was assessed based on the remaining amount of eye drop. One drop of the solution was approximately 0.04 ml, and each child would have used more than 2.4 ml each month. If the child’s remaining eye drops in any bottle exceeded 10% (about 1 ml) of the total amount in each bottle, then their compliance was not good. We carefully checked and recorded the OK lenses, lens case, lens suction holder, and care solutions at each visit. Specifically, the average weekly use of eye drops and wearing of OK lenses was assessed using a paper questionnaire at each follow-up visit. To improve compliance, we adopted the following methods: (1) We explained to the children and their parents the importance of using eye drops and wearing the OK lens correctly daily for myopia control. (2) A WeChat group was set up for all parents of the children, and two colleagues of the research group answered all kinds of questions encountered by the children during the trial. (3) Parents were asked to set a reminder before sleeping for applying eye drops before wearing the OK lens because both procedures had to be performed daily.

### Study procedures

The details of the examination method for the observation items have been published elsewhere and are briefly described here [7, 15]. AL, corneal power, and anterior chamber depth (ACD) were evaluated using a non-contact partial coherence interferometer (IOLMaster; Carl Zeiss Meditec AG, Germany). Five successive measurements were taken on each occasion, and their means were used for analysis. Pupil diameter was measured with an autorefractor (NIDEK, AR-1, Japan) under bright light indoors. The light in the examination room was maintained at constant illumination of 300–310 lx (TES-1332 A illumination photometer). The children had to adapt to ambient light in the examination room for 10 min before the measurement. Three consecutive measurements were performed, and the average values were recorded. The AMP was measured monocularly using the push-up technique. The children wore their fully corrected spectacle prescription and focused on the previous line of best-corrected visual acuity in the right eye while the left eye was occluded. The children were instructed to focus on one letter as the chart moved closer. They were told to keep the letter as clear as possible until they could no longer be held in clear focus. The inverse of the final distance in meters was recorded as the AMP of the child. The AMP was recorded three times, and the average was used for analysis. Discomfort symptoms in the experimental groups were assessed using a written questionnaire during each follow-up visit. Cycloplegic autorefraction was performed using four drops of compound tropicamide eye drops (0.5% tropicamide and 0.5% neo-synephrine) (Santen, Japan) administered to both eyes at an interval of 10 min [26, 27]. Ten minutes after the last drop, cycloplegic autorefraction was measured thrice using an autorefractor (Topcon RM 8000 A, CA), and the readings, all within a difference of 0.25 D, were averaged for analysis. SER was calculated as the sphere plus half of the cylindrical power.

### Sample size and data analysis

The sample size was calculated based on the results of previous studies [16–18]. We assumed that 90% power was required to detect at least 0.10 mm AL difference between the combination and control groups, with significance at the two-sided 5% level and standard deviation of 0.15 mm. Thus, this cross-over trial required overall 48 participants in the two groups. Considering a dropout rate of 20%, a total of 60 participants would be adequate.

Both eyes were treated and tested, but only right eye data from subjects who completed the 12-month follow-up were used for the analysis. The data was analyzed on a per-protocol basis. That is, subjects with poor compliance were not included in the statistical analysis. Normal distributed continuous variables were expressed as mean ± standard deviation (SD) and evaluated using Student *t*-test. Nonnormally distributed continuous variables were presented as medians with first and third quartiles [Q1, Q3] (95% CI) and evaluated using non-parametric rank-sum test. Categorical variables, such as sex and parental myopia status, were expressed as percentages (%) and evaluated using the chi-square test. Two-factor repeated measures ANOVA were performed for the AL, PD and AMP at each time point with treatment group (combination and control groups), time, and interaction of time and group included in the model setup. Multivariate linear regression analyses were used to compare AL changes between combination and control groups and assess the relationship between age, baseline SER and AL, changes in PD, and axial elongation at the 12-month visit. Statistical significance was set at p < 0.05. All statistical analyses were performed using Empower(R) software (WWW.EMPOWERSTATS. COM, X & Y Solutions Inc., Boston, MA, USA) and R (http://www.R-project.org).

## Results

A total of 60 children (30 in the combination group and 30 in the control group) who had been wearing OK lenses successfully for 2 months were enrolled in this study. No differences were found in the parameters between the groups at enrollment (Table 1). Of the 60 enrolled children, 53 completed the 1-year follow-up examinations, 7 children (11.7%) dropped out, including 3 (10%) and 4 (13.3%) from the combination and control groups, respectively (Fig. 1). There were no significant differences in the parameters at enrollment between the dropout children and those who completed the study (p > 0.05). Of the 7 children who dropped out, 5 children who completed the 1-year follow-up showed poor compliance (8.3%), in detail, 1 children had three bottles, and 1 child had four bottles eye drops with more than 1 ml left in the combination and control groups, respectively. One child in the combination group and 2 child in the control group did not wear OK lenses for more than 1 month.

**Table 1:** Characteristics at enrollment of subjects in the two groups followed for 1 year, mean ± SD or n (%)

Variables	Combination group (n = 27)	Control group (n = 26)
Age (year)		
All	10.07 ± 1.44	9.80 ± 1.64
Age (8.0 to10.0 years)	9.77 ± 1.26 (n = 13)	9.65 ± 1.45 (n = 12)
Age (10.1 to 12.0 years)	10.34 ± 1.58 (n = 14)	9.98 ± 1.89 (n = 14)
Spherical equivalent refraction (SER, D)		
All	-2.81 ± 0.92	-2.81 ± 0.97
SER (-1.00 to -2.50D)	-2.61 ± 1.04 (n = 14)	-2.76 ± 1.24 (n = 13)
SER (-2.51 to -4.00 D)	-3.10 ± 0.68 (n = 13)	-2.84 ± 0.71 (n = 13)
Body mass index (kg/m2)	18.94 ± 4.12	17.48 ± 3.40
Age at myopia diagnosis (year)	8.80 ± 1.50	8.84 ± 1.66
Age at first wearing of glasses (year)	9.06 ± 1.62	8.83 ± 1.59
SER progression 1 year before study enrollment (D)	-0.72 ± 0.48	-0.77 ± 0.38
Corneal curvature (D)	42.84 ± 1.38	42.88 ± 1.25
Corneal astigmatism (D)	1.07 ± 0.48	1.23 ± 0.44
Anterior chamber depth (mm)	3.59 ± 0.42	3.70 ± 0.25
Intraocular pressure (mmHg)	16.96 ± 3.13	16.47 ± 2.43
Pupil diameter (mm)	5.58 ± 1.48	5.72 ± 1.19
Accommodative amplitude (D)	16.11 ± 3.67	15.99 ± 4.03
Axial length (mm)	24.79 ± 0.72	24.64 ± 0.79
Male, n (%)	10 (37.04%)	15 (57.69%)
Heredity		
- - (neither parent myopic)	2 (7.41%)	5 (20.00%)
+ - (one parent myopic)	11 (40.74%)	9 (36.00%)
+ + (both parents myopic)	14 (51.85%)	11 (44.00%)

Note: Combination group: combination of OK lens and 0.01% atropine eye drops; Control group: combination of OK lens and placebo

**Fig. 2:**
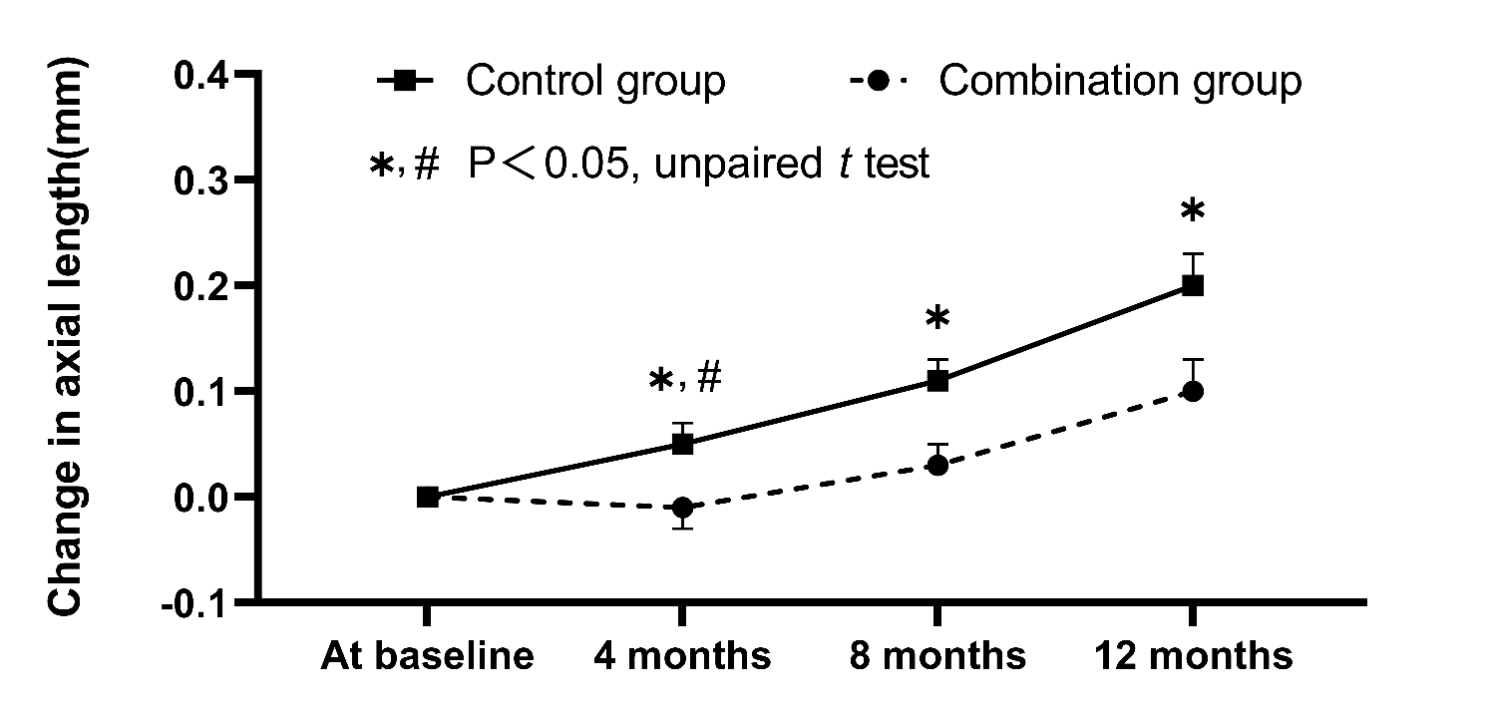
Changes in axial length over time in the combination and control groups.  and  represent standard errors. * Represents a significant difference in axial elongation between two groups at 4-, 8- and 12-month (All p < 0.05). # Represents a statistically significant difference in AL change between two groups only in the first 4 months (p = 0.04)

During the pre-study period of about 3.5 months after enrollment, the increase in AL did not differ significantly between the combination (0.05 ± 0.11 mm) and control groups (0.06 ± 0.07 mm) (p = 0.56). Figure 2 shows the changes in axial length over time in the combination and control groups. In detail, the increase in AL was smaller in the combination group than the control group in the first 4 months (median [Q1, Q3] (95% CI), -0.01 mm [-0.07, 0.05] (-0.06, 0.04) vs. 0.04 mm [0.00, 0.10] (0.02, 0.09)), the middle 4 months (0.04 mm [-0.01, 0.08] (0.01, 0.06) vs. 0.07 mm [0.04, 0.09] (0.03, 0.09)), and the last 4 months (0.05 mm [0.03, 0.10] (0.04, 0.09) vs. 0.07 mm [0.03, 0.14] (0.04, 0.12)), but the difference in change in AL between the two groups was statistically significant only in the first 4 months (p = 0.04) (Fig. 2). Table 2 showed the mean and standard deviation of AL at baseline and 4-, 8-, and 12-month visits, respectively. In comparison of the changes in AL from baseline across 12-month visits between the two groups, statistically significant differences were found between groups (repeated measures ANOVA, time, and treatment group as factors; p = 0.02). In addition, significant changes in AL over time were found within the two groups (time; p < 0.001). Over 1-year period, the overall axial elongation was 0.10 ± 0.14 mm and 0.20 ± 0.15 mm (p = 0.01) in the combination and control groups, respectively. In multivariate linear regression analysis, after adjusting for potential confounders (sex, age, and SER at baseline), the axial elongation was significantly slower in the combination group than in the control group (standard β = -0.10, 95% confidence interval: -0.17 to -0.03, p = 0.02). In multivariate linear regression analyses after adjusting for sex, SER, and corneal curvature, age had no significantly correlation with axial elongation in the combination and control groups (all p > 0.05), baseline SER and AL (adjusting for sex, age, and corneal curvature) were also not associated with axial elongation in the two groups (all p > 0.05).

**Table 2:** Measurements of axial length, pupil diameter and accommodative amplitude over time in two groups

	Combination group (n = 27)	Control group (n = 26)	P value		
			Group	Time	Group * Time
Axial length (mm)			0.27	<0.001	0.02
Baseline	24.84 ± 0.76	24.70 ± 0.74			
4 months	24.83 ± 0.70	24.75 ± 0.71			
8 months	24.87 ± 0.75	24.81 ± 0.69			
12 months	24.94 ± 0.79	24.90 ± 0.80			
Accommodative amplitude (D)			0.65	0.08	0.64
Baseline	16.70 ± 3.47	16.67 ± 3.99			
4 months	15.00 ± 2.61	15.47 ± 2.42			
8 months	15.22 ± 2.73	15.52 ± 2.63			
12 months	15.12 ± 3.19	15.38 ± 2.92			
Pupil diameter(mm)			0.61	0.06	0.04
Baseline	5.72 ± 1.42	5.87 ± 1.39			
4 months	6.19 ± 1.30	5.71 ± 1.32			
8 months	6.14 ± 1.26	5.78 ± 1.40			
12 months	6.11 ± 1.45	5.84 ± 1.60			

Note: Two-factor repeated measures ANOVA were performed for the axial length, accommodative amplitude, and pupil diameter at each time point with treatment group (combination and control groups), time, and interaction of time and group

Compared with the enrollment, no statistically significant changes in AMP and PD were observed in the combination and control groups after wearing OK lens for 2 months (all p > 0.05). Table 2 showed the measurement of AMP and PD over time in the two groups. Compared with baseline, there was no significant interaction between time and group in AMP (repeated measures ANOVA, time and treatment group as factors; p = 0.64), the AMP tended to decrease after wearing OK lens but the changes were statistically insignificant in the two groups at the 4-month visit (all p > 0.05). The AMP in the two groups remained stable from 4 months to 12 months. Compared with baseline, there was significant interaction between time and group in PD (repeated measures ANOVA, time and treatment group as factors; p = 0.04), the PD in the combination group significantly increased by 0.45 mm [0.20, 0.68] at 4 months (p < 0.001), and then remained stable from 4 months to 12 months, while the PD in the control group remained stable from baseline to 12 months. In multivariate linear regression analyses, after adjusting for sex, age, corneal curvature, and SER, the change in PD was negatively associated with axial elongation in the combination group (standard β = -0.03, 95% confidence interval: -0.06 to -0.01, p = 0.03).

## Adverse events

After using eye drops, four (13.3%, combination group) and three children (10%, control group) were mildly photophobic in bright sunlight, while no other discomfort in normal indoor or daily outdoor light was experienced. Photophobia disappeared in 6 children after applying eye drops for 1 to 4 weeks. For one child in the combined group, photophobia did not regress completely but showed slight improvement after six months. Photophobia was resolved by wearing sunglasses or sunhats during outdoor activity. One child in the combination group had mild near-vision blur after treatment with 0.01% atropine for 1 week. None of the children in either group showed any other discomfort symptoms. One child in the control group developed infiltrative keratitis after wearing the OK lens for one month, however, ocular health was fully restored after OK lens discontinuation and treatment with topical antibiotics for one week.

## Discussion

This randomized, double-blinded, placebo-controlled one-year trial showed that the average axial elongation in myopic children was significantly slower in the OK lens combined with 0.01% atropine group than in the OK lens alone group, and mainly in the first 4 months, on the premise that they had already worn the OK lens for two months before 0.01% atropine or blank solvent were introduced in the two groups.

Two randomized, not-blinded studies in Japan and Hong Kong China have described the additive effect of 0.01% atropine and OK lens in slowing axial elongation [16–18]. A one-year trial in Japan including children aged 10.6 years (8 to 12 years old) with an average SER of -2.88 D (-1.00 to -6.00 D) showed that the combination of OK and 0.01% atropine was more effective in slowing axial elongation than OK lens alone (0.09 mm vs. 0.19 mm) [16, 17]. A one - year Hong Kong China trial evaluated children aged 9.0 years (6 to 11 years old) with average SER of -2.75 D (-1.00 to -4.00 D), and found that the axial elongation was slowed by 0.09 mm (0.07 mm vs. 0.16 mm) in combination group compared to monotherapy group with OK lens [18]. In a retrospective two-year study in Taiwan [19], Wan et al. also found improved myopia control by combining OK lenses with 0.025% atropine in children with an average age of 10.35 years and an average SER of 4.58D, compared with OK lenses alone (0.32 mm vs. 0.41 mm per year). In the current trial, axial elongation was slower by 0.10 mm in the combination group than the OK lens alone group, the one-year additive effect observed in the current study was similar to the above three studies. In addition, the additive effect was more obvious in the first 4 months, and the combination effect was significantly weakened in the middle and last 4 months. One 1-year and one 2-year study found that additive effect was only within the first 6 months and 12 months, respectively [17, 18]. One possible reason may be that the eyes adapted to 0.01% atropine, resulting in the additive effect wearing off with time [18].

The mechanisms by which the combination of OK lens and 0.01% atropine was more effective in slowing axial elongation than OK lens alone remain uncertain. 0.01% atropine in the combination group significantly increased PD in children, which may facilitate the effect of OK lens to slow axial elongation through both pharmacological and optical mechanisms [16, 18]. Studies from different countries have shown that moderate- and low-concentration atropine (e.g., 0.025%, 0.05%) may effectively and safely slow the progression of myopia in children [5–7, 13, 28]. It may have biochemical effects on the retina or sclera, which in turn affect remodeling of the sclera [29], or may increase collagen cross-linking with the sclera by increasing ultraviolet exposure (secondary to pupil dilation), thereby limiting scleral growth [30]. As described previously, OK lens may control myopia progression and slow axial elongation by increased peripheral myopic defocus with increased higher-order aberration [8–10, 31], whereas the increased higher-order aberration through the corneal epithelial redistribution [24, 32]. In theory, an enlarged PD could increase the exposure of relative peripheral myopic defocus on the retina of OK lens-treated eyes. Similar to Tan’s study [18], the current study found that the change in PD had a negative correlation with axial elongation in the combination group. However, we did not measure peripheral refraction or higher-order aberrations in this study. Further analyses are warranted to clarify whether there is an association between changes in optical metrics such as peripheral defocus and axial elongation to provide further understanding of the mechanism for myopia control.

Increase in PD is one of the most important side effects of low-dose atropine eye drops [5–7, 15]. Our study found that 0.01% atropine had a minimal impact on PD in the combination groups. Application of 0.01% atropine alone in myopic children resulted in initial increase in PD, which then remained stable. The AMP increased by approximately 0.60 D from enrolment to wearing the OK lens for 2 months (baseline) and then decreased by approximately 1.50 D from baseline to wearing the OK lens for another 12 months in the two groups, but both the changes were not statistically significant. Overall, AMP levels did not change in either group. The changing trend of AMP rising first and then declining in children with myopia wearing the OK lens was consistent with Song et al. ’s study [33]. However, Yang reported that the AMP in the OK lens group increased as wearing time extended [34], the changing speed was faster after wearing the OK lens for 1 to 6 months, and then slowed down. Several studies, including our previous studies [5–7, 15], have found that AMP is significantly reduced after using low-dose atropine. However, no significant changes were found in AMP in either the combination group or the OK lens alone group, which was consistent with the study by Tan et al. [18]. There was a similar proportion of ocular symptoms, such as photophobia, occurring after using low-dose atropine or blank solvent for 1 to 4 weeks. Eventually, photophobia disappeared in all affected children except one child in the combination group. Photophobia may show individual differences regardless of age, sex, myopic degree, and other parameters [35].

The strengths of this study lie its randomized, double-blinded, placebo-controlled trial design. However, our study had several limitations. First, the sample size was calculated based on crossover design. However, this study only reported pre-crossover results, so the sample size was small, which partly limits the statistical power of this explorative study. However, the current sample size could satisfy the statistical power of the AL differences between two groups and was sufficient to draw the present conclusions. Second, the current first year results are based on the parallel control data before the crossover (phase 1), and the second year (phase 2) results of swapping eyedrops in the two groups will be reported next. Third, lack of a real control group which subjects using spectacles alone and a treatment arm where only using 0.01% atropine. An 0.01% atropine-only group made us know that the better results in the combination group is solely because of atropine or other reasons. However, the purpose of current study was to investigate whether there is an additive effect of combining 0.01% atropine with OK lens, using OK lens alone as comparator. The absence of an 0.01% atropine-only group did not affect the investigation of a combined treatment. Moreover, the measurement of peripheral refraction and higher-order aberration is required to clarify the mechanism of the additive effects of OK and atropine 0.01% ophthalmic solution. Additionally, 0.05% atropine was more effective than 0.01% atropine in the LAMP study [7]. Therefore, further studies including higher atropine concentrations combined with OK lens and a treatment arm where only using low concentration atropine should be conducted in the future.

## Conclusion

Our preliminary one-year findings showed that the axial elongation of the myopic children was significantly slower in the OK lens combined with 0.01% atropine than in the OK lens alone, and mainly in the first 4 months of the combination therapy.

## Data Availability

The datasets analyzed in the current study are available from the corresponding author for reasonable requests.

## Abbreviations

OK: Orthokeratology
SER: Spherical equivalent refraction
AL: Axial length
PD: Pupil diameter
AMP: Accommodative amplitude
